# Root Branching Is Not Induced by Auxins in *Selaginella moellendorffii*

**DOI:** 10.3389/fpls.2019.00154

**Published:** 2019-02-20

**Authors:** Tao Fang, Hans Motte, Boris Parizot, Tom Beeckman

**Affiliations:** ^1^Department of Plant Biotechnology and Bioinformatics, Ghent University, Ghent, Belgium; ^2^VIB Center for Plant Systems Biology, Ghent, Belgium

**Keywords:** root branching, *Selaginella*, evolution, auxin, bifurcation

## Abstract

Angiosperms develop intensively branched root systems that are accommodated with the high capacity to produce plenty of new lateral roots throughout their life-span. Root branching can be dynamically regulated in response to edaphic conditions and provides the plants with a soil-mining potential. This highly specialized branching capacity has most likely been key in the colonization success of the present flowering plants on our planet. The initiation, formation and outgrowth of branching roots in Angiosperms are dominated by the plant hormone auxin. Upon auxin treatment root branching through the formation of lateral roots can easily be induced. In this study, we questioned whether this strong branching-inducing action of auxin is part of a conserved mechanism that was already active in the earliest diverging lineage of vascular plants with roots. In Selaginella, an extant representative species of this early clade of root forming plants, components of the canonical auxin signaling pathway are retrieved in its genome. Although we observed a clear physiological response and an indirect effect on root branching, we were not able to directly induce root branching in this species by application of different auxins. We conclude that the structural and developmental difference of the Selaginella root, which branches via bifurcation of the root meristem, or the absence of an auxin-mediated root development program, is most likely causative for the absence of an auxin-induced branching mechanism.

## Introduction

Roots, the hidden half of plants, anchor the plant body to the ground and absorb water and nutrients. The evolution of roots has been a very important innovation for plants to successfully colonize the terrestrial environment over about 400 million years ago (Raven and Edwards, 2001). The branching capacity of roots was of utmost importance in the colonization of plants, as it allows, beside a strong anchor, exploration of the soil and foraging of nutrients and water.

In angiosperms such as *Arabidopsis*, roots branch by the formation of lateral roots, which initiate from specialized pericycle cells. Auxin, a powerful plant growth regulator, plays a key role in this biological process by stimulating and activating pericycle cells to specify lateral root founder cells (Himanen et al., 2002; Dubrovsky et al., 2008; De Rybel et al., 2010; Möller et al., 2017). The importance of auxin signaling in promoting lateral root formation through several Aux/IAA-ARF modules has been well studied (Fukaki et al., 2002; De Rybel et al., 2010; De Smet et al., 2010; Moreno-Risueno et al., 2010; Goh et al., 2012; Du and Scheres, 2018). Accordingly, polar auxin transport inhibitors, such as 1-n-naphthylphthalamic acid (NPA) and 2,3,5-triiodobenzoic acid (TIBA), were found to inhibit lateral root branching through blocking auxin efflux (Karabaghli-Degron et al., 1998; Reed et al., 1998; Casimiro et al., 2001; Himanen et al., 2002). In addition to polar auxin transport inhibitors, cytokinin can also inhibit lateral root initiation possibly through a separate signaling pathway (Li et al., 2006; Chang et al., 2013). Similar to lateral root initiation, auxin signaling and auxin transport are crucial in root meristem and lateral root meristem establishment (Sabatini et al., 1999; Benkova et al., 2003; Blilou et al., 2005; Dello Ioio et al., 2008; Du and Scheres, 2017, 2018).

In contrast to lateral branching roots, lycophyte roots branch via bifurcation of the root tip, also called dichotomous branching (Webster and Steeves, 1964; Otreba and Gola, 2011; Gola, 2014). The lycophyte clade is the most ancient clade with rooting plants. Selaginella is an important representative of the lycophytes (Banks, 2009), in particular as it includes *Selaginella moellendorffii*, the first lycophyte with a sequenced genome (Banks et al., 2011). Roots of Selaginella originate from rhizophores, root primordia-bearing organs that develop on stems. The root meristem originates from a single tetrahedral apical stem cell and is anatomically similar to the root meristem in ferns. This meristem is however very different to seed plants (Motte and Beeckman, 2018). Root dichotomous branching occurs through the bifurcation of the meristem, presumably via the activation of two new apical stem cells (Gola, 2014). As such, two new root meristems establish within the root tip (Imaichi and Kato, 1989; Otreba and Gola, 2011).

Auxin signaling and auxin transport are highly conserved within land plants and key components of these pathways are already present in Selaginella (Viaene et al., 2013, 2014; Bennett et al., 2014; Kato et al., 2018; Mutte et al., 2018). In addition, the auxin flow toward the Selaginella root apex (Wochok and Sussex, 1974) suggests an important role of auxin in the root meristem as well. Considering its importance in root initiation and root meristem establishment in many land plants, a role of auxin in the initiation of dichotomous root branching in Selaginella could be anticipated as well. This putative role was previously suggested based on heavily branching roots upon auxin treatment in *Selaginella kraussiana* (Sanders and Langdale, 2013), but to our knowledge, no data on the induction of root branching are available up to date. To fill this gap, we report here on the effect of auxin on root initiation and dichotomous branching in *Selaginella moellendorffii*. Surprisingly, we found that auxin does not induce the root bifurcation itself.

## Materials and Methods

### Plant Material and Growth Conditions

**Figure 1:**
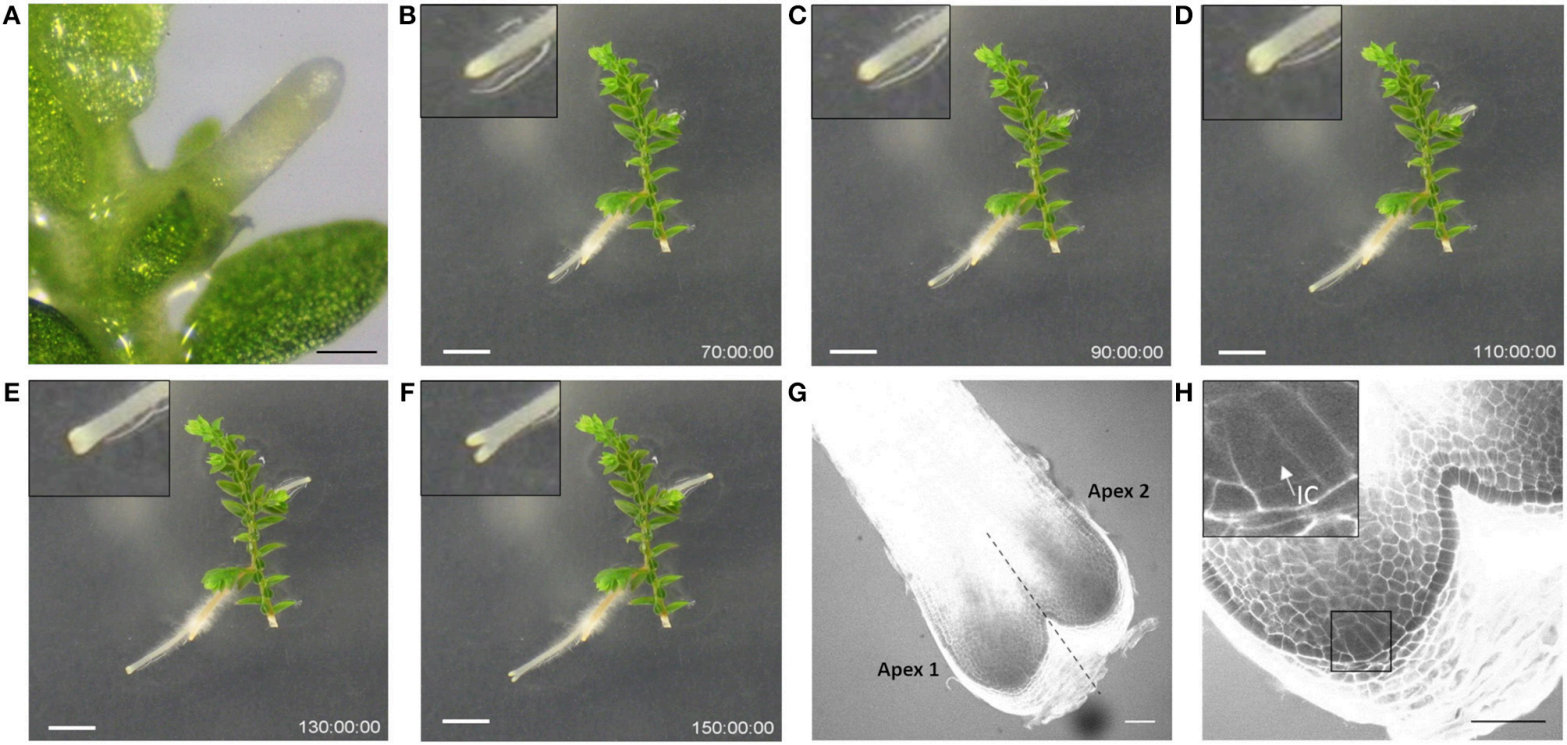
Rhizophore and dichotomous root branching in Selaginella. **(A)** Rhizophore emerged from the stem. **(B–F)** Frames from Video S1 showing the process of dichotomous root branching. Newly branched roots as in **(D,E)** were used as starting material in the branching experiments. The time is indicated in hours. Scale bars: 1 mm. **(G)** Representative confocal image of a newly branched root. **(H)** Magnification of apex 1 in **(G)** shows one single IC. The inset is a magnification of the square. IC, initial cell. Scale bars: 50 μm.

*Selaginella moellendorffii* (Selaginella) plants were obtained from the lab of Jo Ann Banks at Purdue University. Plants are routinely propagated on sterile half-strength Murashige and Skoog (1/2MS) medium (Duchefa Biochemie) supplemented with 0.8% (w/v) agar, pH 5.8, in Sterivent boxes (Duchefa Biochemie) in a growth room at 24°C with light intensity 20.25–43.2 μmol/m^2^/s (cool white fluorescent lamps) and regime of 16 h light and 8 h dark. To induce rhizophores or roots, shoot apical segments, presenting two branches (further referred as explants), were transferred into Petri dish plates with 1/2MS. After a few days, rhizophores and roots started to emerge, as illustrated in Figure 1 and Video S1 showing growth of an explant from 8 days post transfer onwards.

To test the promotive/inhibitory effect of auxin compounds as well as potential inhibitors on the root bifurcation, explants incubated for 12 days on 1/2MS were transferred to the treatment medium and only roots that just underwent a new branching event were used for analysis. For this purpose, all roots were preliminary screened at 11 and 12 days of incubation with a stereomicroscope. Roots that bifurcated between day 11 and day 12 were annotated as newly branched roots (Figure 1D or Figure 1E). Microscopic analysis of these roots showed that the newly formed tips never contained two meristems (*n* = 58), i.e., the next dichotomous branching was not initiated yet (Figures 1G,H). After transfer to the treatment medium, each root tip was observed daily with a stereomicroscope to evaluate bifurcation. The branching percentage was calculated as the number of bifurcated apices divided by the total number of root apices coming from newly branched roots. The number of branching events in a period of 13 days was counted per root apex coming from a newly branched root. In case of indole-3-acetic acid (IAA) treatments, yellow plastic sheets covering the plates were used to prevent IAA degradation from light.

### Root Morphology

Explants or roots were subjected to daily stereomicroscopic observation to record the number of new emerging rhizophores and bifurcating roots. To determine root length elongation, the Petridish plates were scanned with a flatbed scanner (EPSON Expression 11000XL) and the length of the root segment between two branching sites was measured with ImageJ software (Abramoff et al., 2004). The elongation rate was calculated by dividing the length between two branching sites by the time in days between the two branching events.

### Microscopy

Selaginella root tips were first fixed in 50% methanol and 10% acetic acid and after clearing subjected to a modified pseudo-Schiff propidium iodide staining as described previously (Truernit et al., 2008). Analysis was done with a Zeiss LSM5 Exciter confocal microscope with an argon ion laser at 488 nm as the excitation source and a detection filter at 505 nm. For all samples, z-stacks were taken to ensure the possible detection of meristematic regions in different planes.

## Results

### Auxins Do Not Affect the Formation of Root-Bearing Rhizophores in Selaginella

**Figure 2:**
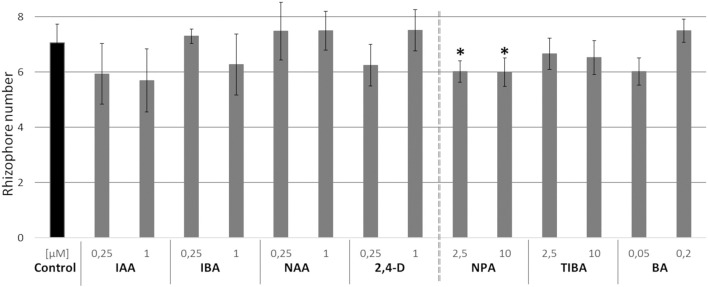
Effects of auxins, polar auxin transport inhibitors and a cytokinin on rhizophore emergence. Selaginella explants with newly branched roots were incubated with dimethyl sulphoxide (DMSO) or different concentrations of treatments for 13 days: indole-3-acetic acid (IAA), indole-3-butyric acid (IBA), 1-naphthaleneacetic acid (NAA), 2,4-dichlorophenoxyacetic acid (2,4-D), naphthylphthalamic acid (NPA), 2,3,5-triiodobenzoic acid (TIBA) or 6-benzylaminopurine (BA). Number of rhizophores emerging post treatments was recorded on 13 d post treatments. Error bars represent SD. *n* (number of plates) = 4 (except for 10 μM TIBA, *n* = 3) with on average 5 explants per plate. ^*^represents *p*-value ≤ 0.05 (Kruskal-Wallis test).

In *Selaginella moellendorffii* (Selaginella), new roots are derived from rhizophores, root-like organs forming on the stem (Figure 1A). In accordance with the positive effect of auxin on adventitious rooting in seed plants, an auxin-dependent effect on the formation of new rhizophores in Selaginella might be anticipated as well. In order to evaluate this putative effect, we investigated the effect of auxins on the formation of rhizophores on Selaginella shoot explants. Hitherto, Selaginella shoot explants of approximately 1 cm were isolated from *in vitro* growing plants and transferred to growth media with different auxins. The number of rhizophores on explants after 13 days of auxin treatments does not significantly differ from the control (Figure 2). Thus, auxins do not promote the formation of rhizophores in Selaginella. Consistently, treatments with auxin transport inhibitors or a cytokinin, which mostly work antagonistically toward auxin in seed plants, did not or only in a limited extent affect rhizophore formation (Figure 2). NPA treatments showed a significant but very modest decrease in rhizophores, indicating rather an indirect role of auxin and auxin transport during rhizophore establishment and emergence.

### Auxins Affect Root Development in Selaginella

In Selaginella, roots branch dichotomously (Figures 1B–H, Video S1). To examine the effect of auxins on root development, shoot explants were first isolated and incubated on hormone-free growth medium to allow the spontaneous formation of rhizophores and roots. To standardize the starting material, explants with a root that recently bifurcated were selected and transferred to an auxin-containing medium. Microscopic inspection of newly formed root tips after bifurcation shows that these apices, without exception, only contain one single meristem. We were able to clearly recognize the described tetrahedral initial cell (IC) and its derivatives form the root meristem in root anatomy of Selaginella (Figures 1G,H). The presence of recently branched root tips in the starting material was thus crucial to dispose of a clear definable stage 0 that can be used for all the treatments.

**Figure 3:**
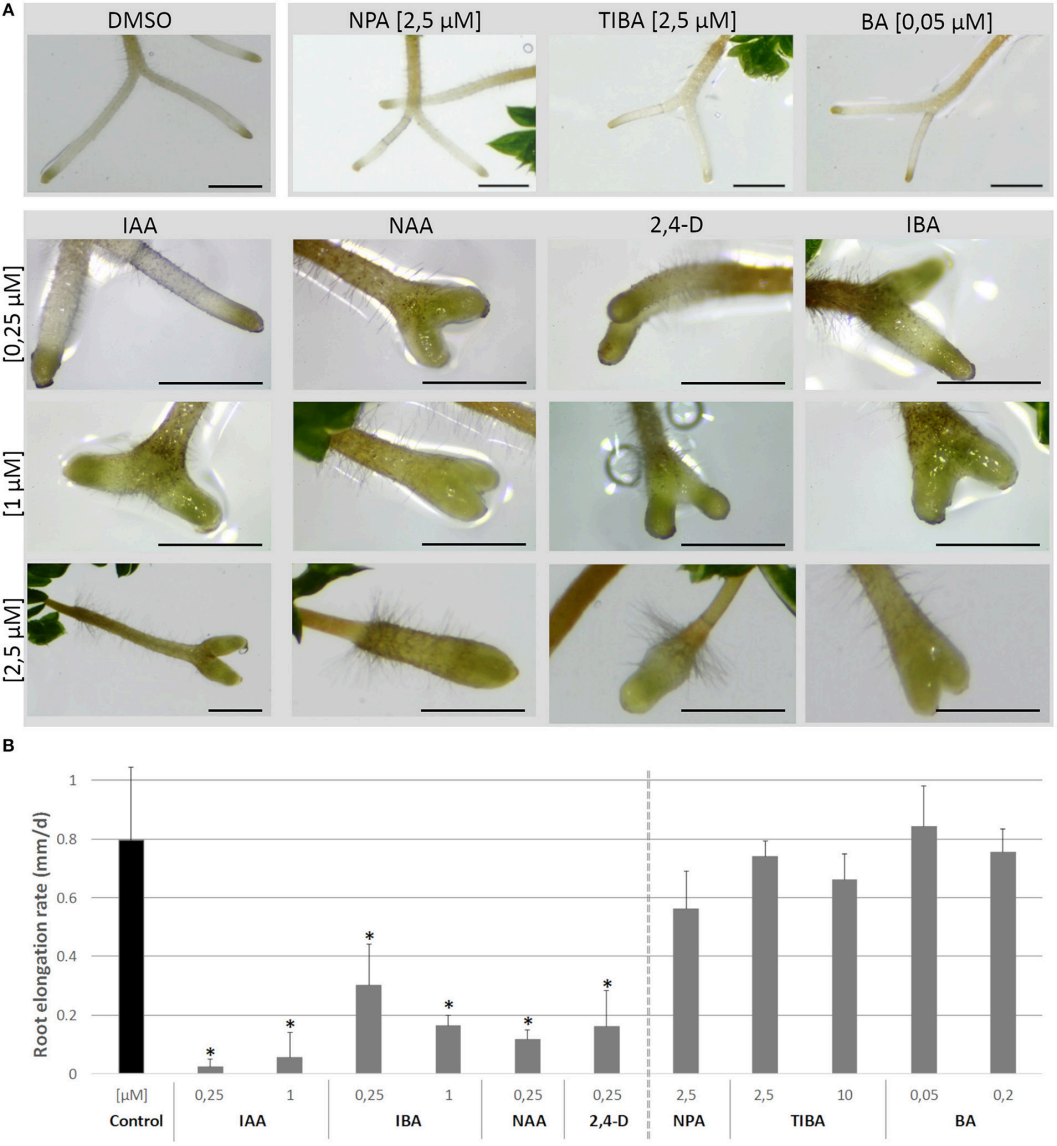
Root growth after treatments with auxins, NPA, TIBA, and BA. **(A)** Explants with newly branched roots were treated with different concentrations of auxins, auxin transport inhibitors or the cytokinin BA for 4 days and root morphology was observed. Scale bars: 1 mm. **(B)** shows the effects of treatments on root elongation rate over the time frame between two branching events. Data is not presented for the treatments that severely inhibited branching or obstructed the observation of possible branching events. Error bars represent SD. *n* (number of plates) = 4 (except for 10 μM TIBA, *n* = 3) with on average 10 root samples per plate. ^*^represents *p*-value ≤ 0.05 (Kruskal-Wallis test).

**Figure 4:**
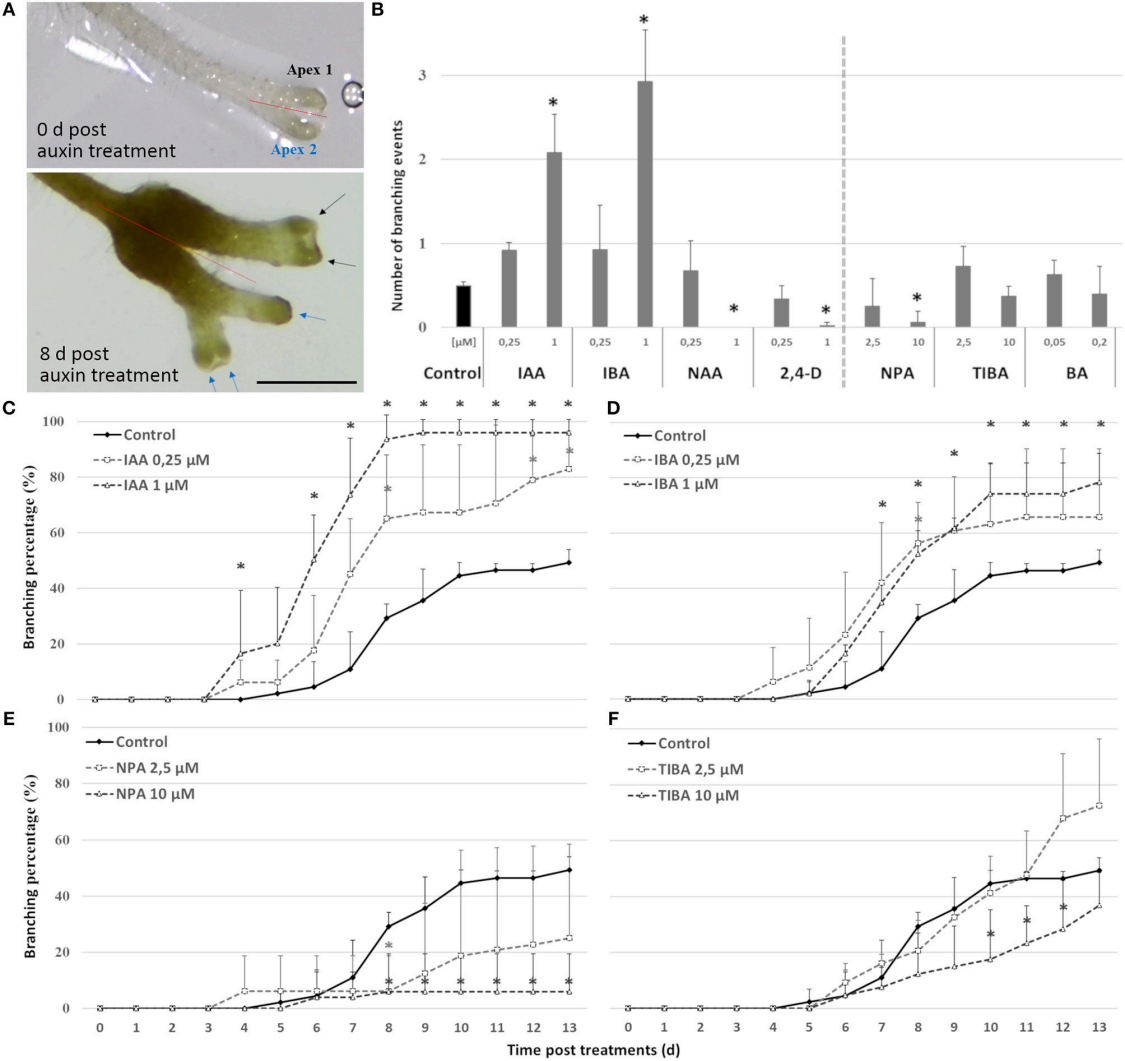
Effect of auxins, polar auxin transport inhibitors and a cytokinin on Selaginella dichotomous root branching. Selaginella explants with newly branched roots were incubated with different concentrations of auxins, auxin transport inhibitors, or the cytokinin BA. **(A)** Selaginella root on 0 and 8 days after IAA (2.5 μM) treatment. Arrows of different colors indicate branching derived from different apices. Scale bar: 1 mm. **(B)** Number of branching events per root tip after 13 days. **(C–F)** Percentage of root tips that branched during 13 days of treatments. Error bars represent SD. *n* (number of plates) = 4 (except for 10 μM TIBA, *n* = 3) with on average 10 root samples per plate. ^*^represents *p*-value ≤ 0.05 (Kruskal-Wallis test).

**Figure 5:**
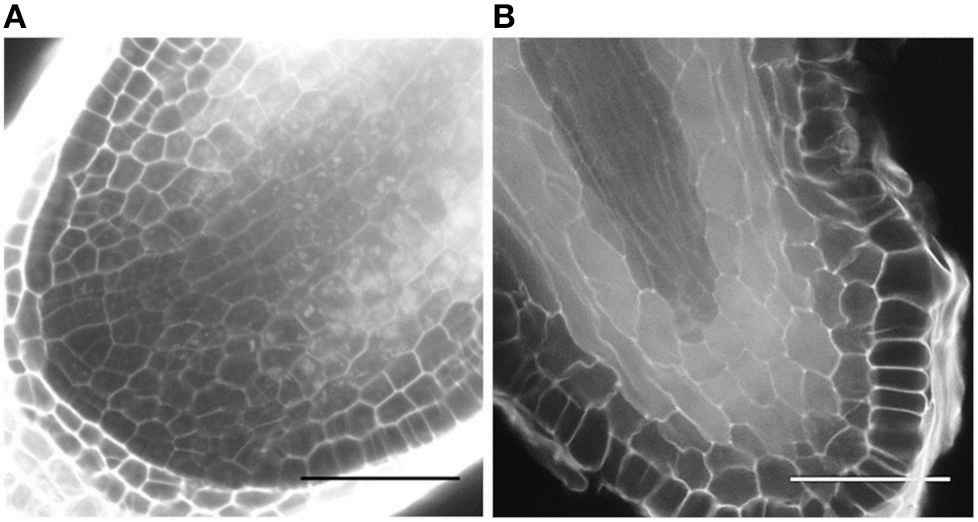
Selaginella root meristem of **(A)** a DMSO-treated root or **(B)** a root treated with 25 μM NPA for 7 days. Scale bars: 50 μm.

On the short term, the auxin treatments, especially at higher concentrations, inhibited root elongation, caused thickening of the root tips, induced callus-like tissue and stimulated root hair elongation (Figure 3). The root elongation rate, calculated between two consecutive branching events, was severely inhibited even at low auxin concentrations. In contrast, the auxin transport inhibitor TIBA and a low concentration (2.5 μM) of NPA showed no or a limited effect on the root elongation and morphology. Only high concentrations (10 μM; 25 μM) of NPA did more severely affect the root morphology (Figure S1). None of the treatments induced laterally positioned root branches, showing that the absence of lateral roots in Selaginella is not due to a limited auxin availability. There was, however, a positive effect on the bifurcation of roots. In accordance to the previous observations of (Sanders and Langdale, 2013), auxin treatment resulted in more branching and a higher branching frequency (Figures 4A,B). When we followed the first branching of individual root tips, in particular IAA and IBA induced much more root tips to bifurcate (Figures 4C,D). NAA and 2,4-D, even at a concentration of 0.25 μM, and all auxins at higher concentrations induced callus, which obstructed the observation of possible branching events (Figure 3A, Figure S2). The auxin transport inhibitors had in most cases no or only a weak effect on the branching (Figures 4B,E,F). Only at higher NPA concentrations, we noticed a strong inhibition of the root branching (Figures 4B,E), with a complete inhibition at 25 μM (data not shown). This however concurred with a growth arrest of the root tip and completely degenerated root meristem (Figure 5). Hence, it seems that auxin transport only affects the root meristem bifurcation when it is almost completely blocked, which in particular disturbs the meristem organization.

Auxin treatments induce only branches after 4 days, which is the same time required to obtain a bifurcation in the hormone-free control. If auxin would be capable in inducing the root meristem bifurcation itself, a much faster induction would have been anticipated.

### Auxins Do Not Induce Root Meristem Bifurcation in Selaginella

**Figure 6:**
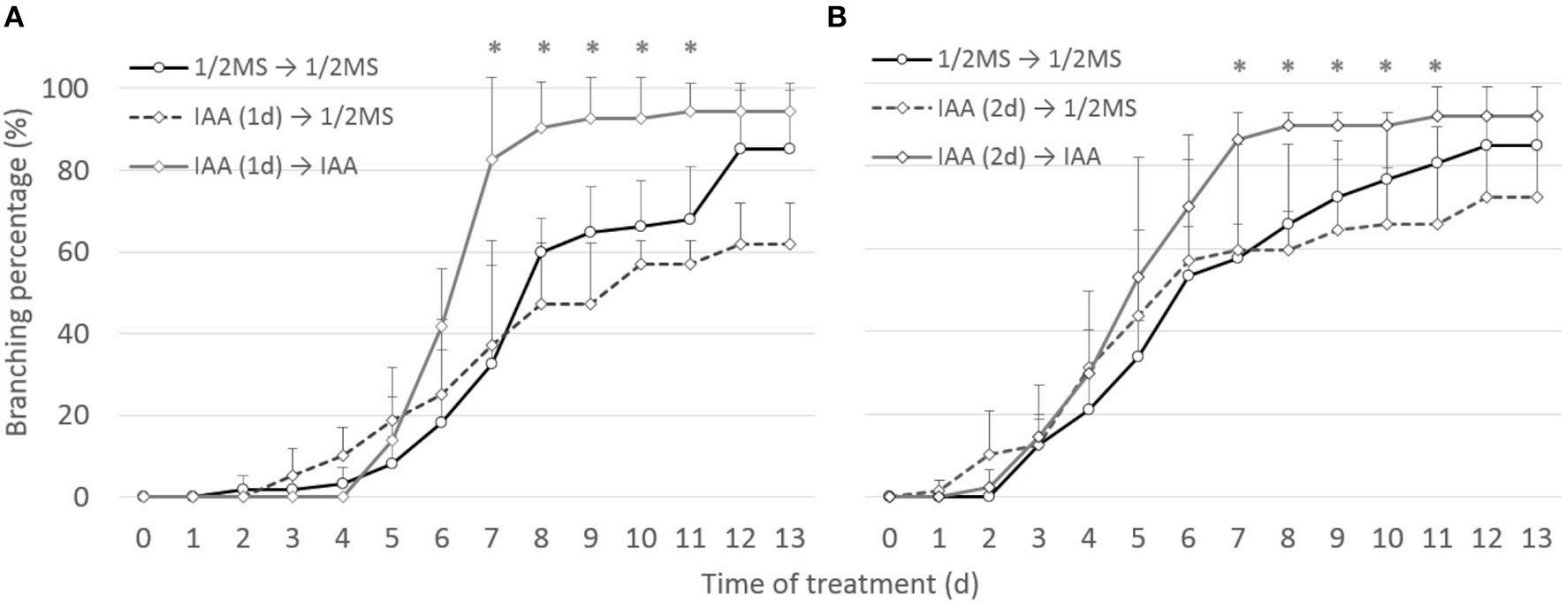
Effect of short auxin treatment on dichotomous root branching. Selaginella explants with newly branched roots were treated with DMSO or 1 μM IAA for 1 **(A)** or 2 **(B)** days. Explants were then transferred to hormone-free or 1 μM IAA-containing media. Error bars represent SD. *n* (number of plates) = 3 with on average 15 root samples per plate. ^*^represents *p*-value ≤ 0.05 (Kruskal-Wallis test).

To ascertain whether the bifurcation-promoting effect of auxin is direct or not, the effect of transient auxin treatments on root branching was assessed. 1 μM IAA was selected for this experiment as it had shown the highest potency in accelerating branching over long treatments. Shoot explants with a newly branched root were transferred to 1 μM IAA for either 1 or 2 days and then transferred back to either hormone-free or IAA medium. In contrast to continuous auxin application (transfer IAA to IAA), none of the transient treatments (IAA to 1/2MS) induced extra root branching compared to the untreated samples (1/2MS to 1/2MS) (Figure 6). This observation strengthens our previous observation that auxins do not induce the bifurcation of the root meristem itself in Selaginella but shorten the timing between two branching events.

**Figure 7:**
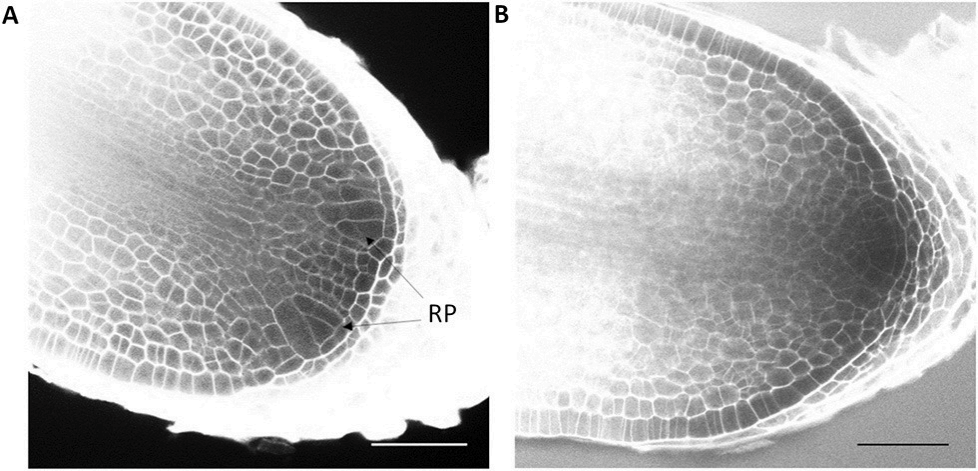
Effect of auxin treatment on bifurcation of root apical meristem. Newly branched roots of Selaginella explants treated by DMSO and IAA were sampled for confocal microscopy daily from 0 to 3 d post treatment. **(A)** A newly bifurcated root meristem 3 days post DMSO treatment. Two new meristems could be clearly recognized. **(B)** A root meristem treated for 3 days with 2.5 μM IAA. RP, root primordium. Scale bar: 50 μm.

To further confirm that auxins do not induce root branching in Selaginella, microscopic analysis at earlier time points was performed using a modified pseudo-Schiff propidium iodide staining and confocal microscopy. We were able to clearly recognize the described Selaginella root anatomy in which one tetrahedral initial cell (IC) and its derivatives form the root meristem (Figures 1G,H). To observe early stages in the meristem bifurcation process, we first collected multiple DMSO-treated root samples at different days after a new branch was formed. Several samples showed an early stage of meristem bifurcation at 3 days (Figure 7A). However, newly branched roots treated for 3 days with 1 μM IAA did not show any meristem bifurcation (*n* = 11). Conversely, a higher auxin concentration hardly affected the induction of meristem bifurcation: either a concentration of 2.5 μM (Figure 7B, *n* = 13) or 5 μM (*n* = 5) IAA yielded only 1 divided meristem after 3 days. Hence, auxins and auxin signaling seem to advance root branching most likely by promoting processes taking place after the early events during root meristem bifurcation and do not directly induce the bifurcation event itself in Selaginella.

## Discussion

Auxin is, in particular regarding to root development, a predominant plant growth regulator with a highly conserved signaling pathway within land plants (Kato et al., 2018; Mutte et al., 2018). Auxins were also reported to induce root cultures in *Selaginella microphylla* (Bandyopadhyay et al., 2013). In accordance with these findings, we did find evidence of a certain level of physiological conservation. Indeed, typical auxin responses such as root hair elongation, callus induction or root elongation inhibition occurred in *Selaginella moellendorffii* as well. In contrast, auxins did not induce root-bearing rhizophores formation nor dichotomous root branching in this species. This is suggestive for the existence of an auxin independent meristem initiation and branching mechanism. Polar auxin transport mechanisms are conserved as well (Viaene et al., 2013, 2014; Bennett et al., 2014; Bennett, 2015) and clearly have an important impact on the growth of Selaginella species (Wochok and Sussex, 1973, 1974, 1975; Sanders and Langdale, 2013; Matsunaga et al., 2017). However, the inhibition of polar auxin transport is not able to block root branching in *Selaginella moellendorffii*, which further supports a potential auxin-independent root branching mechanism. Only high concentrations of NPA resulted in a reduction in root branching, but does not delay the timing in branching, and can be interpreted as a presumably indirect effect.

Sanders and Langdale (2013) previously showed strongly branching root tips upon auxin treatment and hence suggested a role of auxin in this branching. Similarly, we also observed on the long term, in particular with high auxin concentrations, consecutive branches with shorter time intervals. However, as there is no difference in meristem formation, as shown by our microscopic observations, and as transient auxin treatments up to 48 h do not result in an increased branching, the induction of root branching is clearly not directly affected by auxin. Hence, the increased root branching rather seems to be the consequence of an acceleration in development after branching which could have been resulted from shorter cell division cycles.

Possibly, the fundamental structural differences of Selaginella roots (Motte and Beeckman, 2018) are causative for the absence of an auxin-induced branching mechanism. The root meristem and cell divisions within the meristem are patterned differently in lycophytes compared to seed plants and therefore possibly adopted a different controlling program as well. The strong branching capacity of roots in the angiosperms moreover required the development of pluripotent pericycle cells. Although a pericycle layer is present in Selaginella species (Webster and Steeves, 1964), our observations suggests the absence of such pluripotency in the early land plants.

The fossil record shows that Lycophyte roots evolved independently from other clades (Raven and Edwards, 2001; Kenrick, 2002; Friedman et al., 2004; Boyce, 2005, 2010; Doyle, 2013; Hetherington and Dolan, 2018, 2019), but gene expression profiles suggested the presence of a root developmental program in a common ancestor or at least a parallel recruitment of largely the same root developmental program from a common ancestor (Huang and Schiefelbein, 2015). Based on our observations, this common program did, however, not contain the elements for the auxin-mediated meristem induction or root branching, which might only have been evolved later on. Supportive for this, lateral root formation in the fern *Ceratopteris richardii*, an earlier diverging lineage than the angiosperms, occurs also independently from auxin (Hou et al., 2004). Moreover, orthologs of important root meristem or lateral root factors downstream of auxin such as *WOX5* or *LBD16* are not found in the lycophyte clade (Nardmann et al., 2009; Coudert et al., 2013; Zhang et al., 2017). Hence, it seems that at least the root branching mechanisms downstream of auxin were only introduced during evolution after the origin of lycophytes, and further corroborates that roots originated multiple times during evolution.

In conclusion, by providing the first extensive evaluation of the effect of auxins on Selaginella root branching, we showed that root branching is not induced by auxins in this representative of an early diverging lineage of land plants. Despite conserved auxin signaling genes and conserved auxin responses, auxin itself seems to be not important for the root branching program and only acquired this role later during evolution.

## Author Contributions

TF, HM, and BP designed the experiments. TF performed the experiments. TF, HM, BP, and TB analyzed the data and wrote the manuscript. All the authors read and approved the final manuscript.

### Conflict of Interest Statement

The authors declare that the research was conducted in the absence of any commercial or financial relationships that could be construed as a potential conflict of interest.

## Supplementary Material

Figure S1 Root growth after NPA, TIBA and BA treatments. Explants with newly branched roots were treated for 4 days and root morphology was observed. Scale bars: 1 mm.

Figure S2 Effect of applied NAA, 2,4-D and BA on dichotomous root branching. Selaginella explants with newly branched roots were incubated with DMSO or different concentrations of treatments for 13 days: NAA **(A)**, 2,4-D **(B)**, and BA **(C)**. Error bars represent SD. *n* (number of plates) = 4 with on average 10 root samples per plate. ^*^represents *p*-value ≤ 0.05 (Kruskal-Wallis test).

Video S1 Selaginella shoot explant transferred to 1/2MS. The video shows the emergence of rhizophores and the subsequent development of roots from 8 days onwards.
