# Supplementary figures and images for: Root Branching Is Not Induced by Auxins in Selaginella moellendorffii
Source: Front Plant Sci. 2019 Feb 20;10:154. doi: 10.3389/fpls.2019.00154 (PMC6391681; doi:10.3389/fpls.2019.00154)

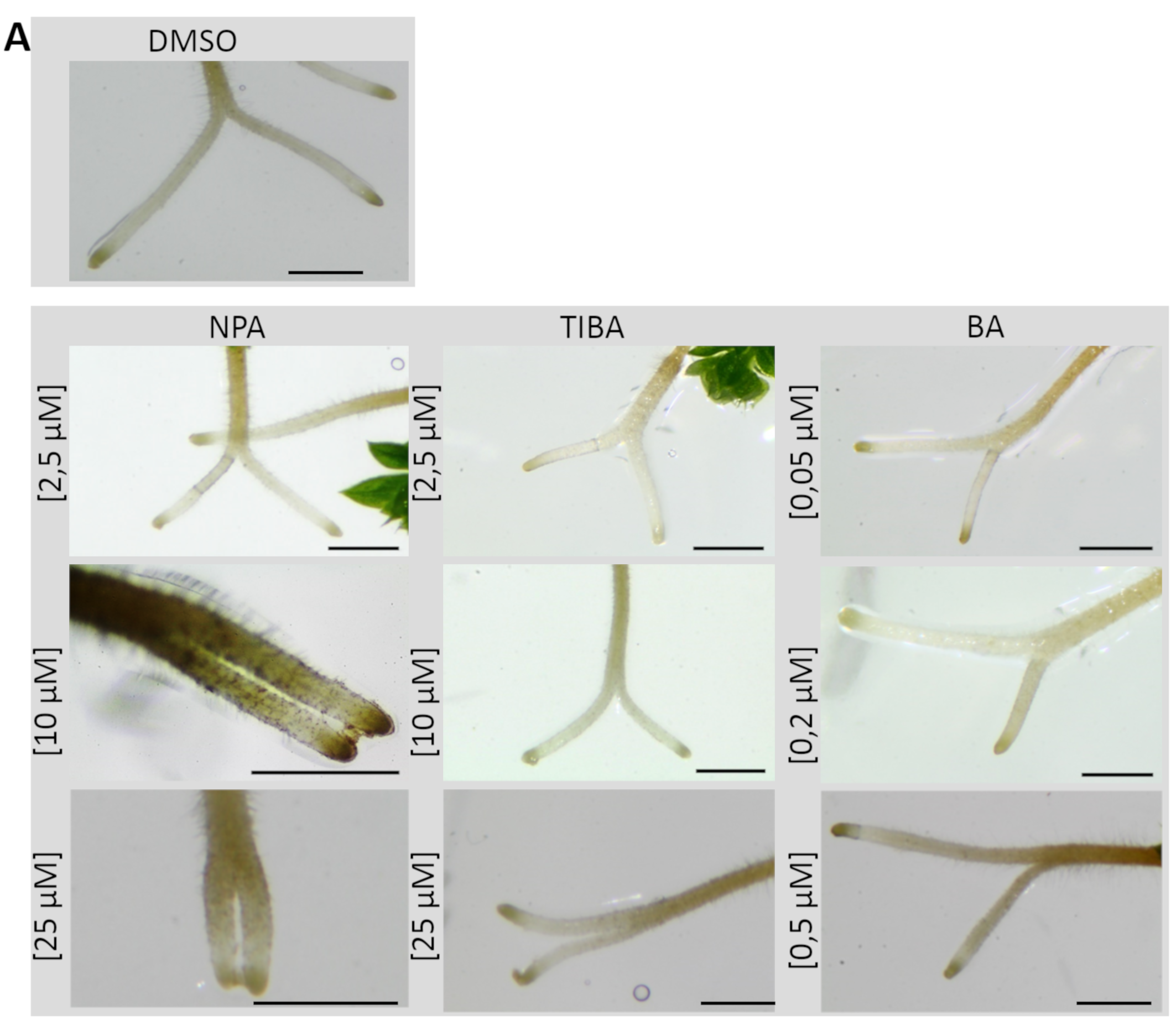

Supplement: Figure S1 — Root growth after NPA, TIBA and BA treatments. Explants with newly branched roots were treated for 4 days and root morphology was observed. Scale bars: 1 mm. [file Image_1.TIF]

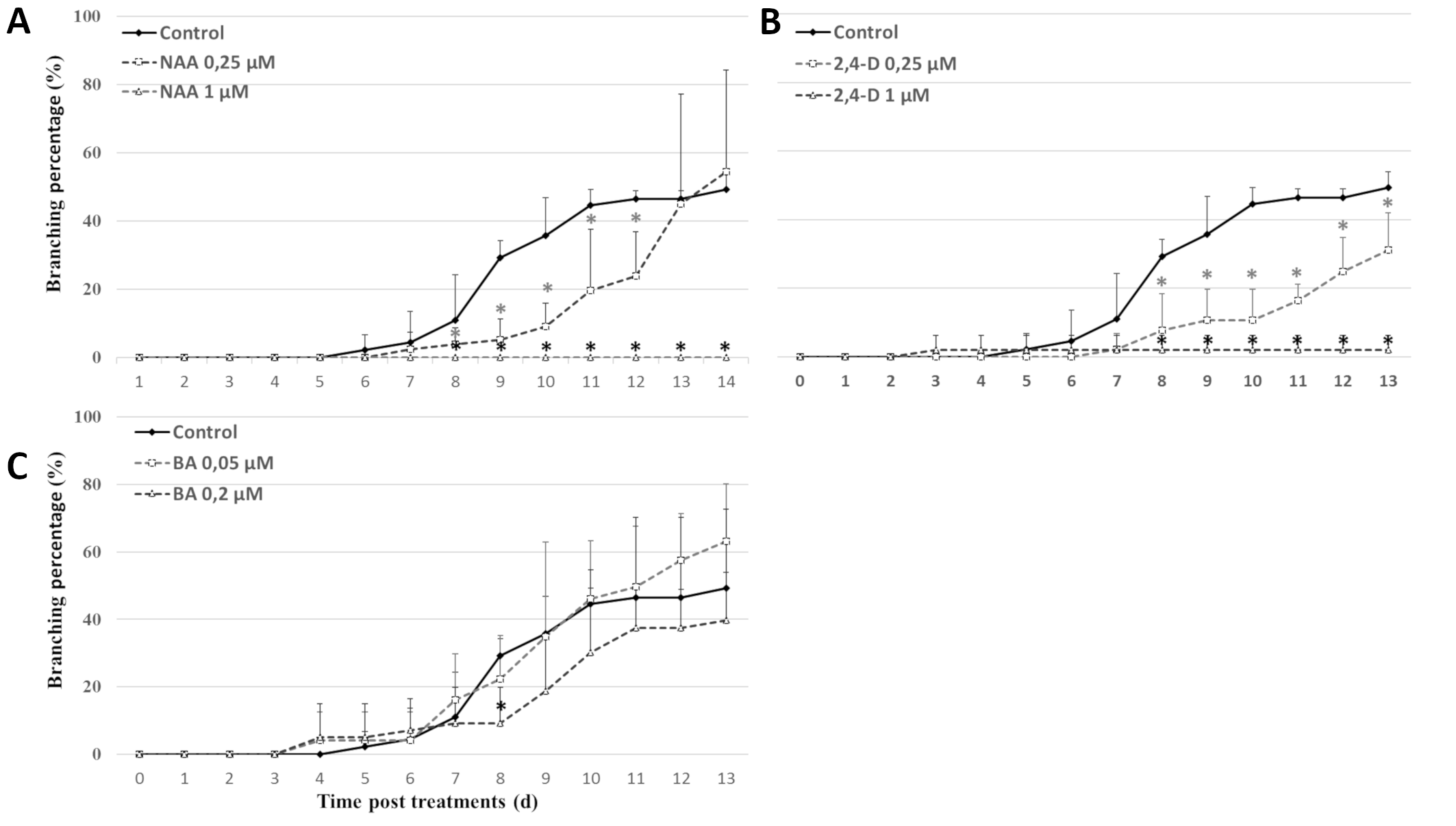

Supplement: Figure S2 — Effect of applied NAA, 2,4-D and BA on dichotomous root branching. Selaginella explants with newly branched roots were incubated with DMSO or different concentrations of treatments for 13 days: NAA (A), 2,4-D (B), and BA (C). Error bars represent SD. n (number of plates) = 4 with on average 10 root samples per plate. *represents p-value ≤ 0.05 (Kruskal-Wallis test). [file Image_2.TIF]
